# Identifying potential therapeutic targets of mulberry leaf extract for the treatment of type 2 diabetes: a TMT-based quantitative proteomic analysis

**DOI:** 10.1186/s12906-023-04140-3

**Published:** 2023-09-04

**Authors:** Lu Shi, Jingkang Wang, Changhao He, Yan Huang, Wanxin Fu, Huilin Zhang, Yongcheng An, Menglu Wang, Ziyi Shan, Huimin Li, Yinglan Lv, Chen Wang, Long Cheng, Hongyu Dai, Yuhui Duan, Hongbin Zhao, Baosheng Zhao

**Affiliations:** 1 Department of Pharmacology of Chinese Materia Medica, School of Chinese Materia Medica, Beijing University of Chinese Medicine, Beijing, 102488 China; 2 Qingdao Municipal Hospital, University of Health and Rehabilitation Sciences, Qingdao, China; 3 College of Life Sciences, Beijing University of Chinese Medicine, Beijing, 102488 China; 4 Dongzhimen Hospital, Beijing University of Chinese Medicine, Beijing, 100029 China; 5 Beijing Research Institute of Chinese Medicine, Beijing University of Chinese Medicine, Beijing, 100029 China

**Keywords:** Type 2 diabetes mellitus, Mulberry leaf, Proteomics, Lipid metabolism

## Abstract

**Background:**

Mulberry (*Morus alba* L.) leaf, as a medicinal and food homologous traditional Chinese medicine, has a clear therapeutic effect on type 2 diabetes mellitus (T2DM), yet its underlying mechanisms have not been totally clarified. The study aimed to explore the mechanism of mulberry leaf in the treatment of T2DM through tandem mass tag (TMT)—based quantitative proteomics analysis of skeletal muscle.

**Methods:**

The anti-diabetic activity of mulberry leaf extract (MLE) was evaluated by using streptozotocin-induced diabetic rats at a dose of 4.0 g crude drug /kg *p.o.* daily for 8 weeks. Fasting blood glucose, body weight, food and water intake were monitored at specific intervals, and oral glucose tolerance test and insulin tolerance test were conducted at the 7th and 8th week respectively. At the end of the experiment, levels of glycated hemoglobin A1c, insulin, free fat acid, leptin, adiponectin, total cholesterol, triglyceride, low-density lipoprotein cholesterol, and high-density lipoprotein cholesterol were assessed and the pathological changes of rat skeletal muscle were observed by HE staining. TMT-based quantitative proteomic analysis of skeletal muscle and bioinformatics analysis were performed and differentially expressed proteins (DEPs) were validated by western blot. The interactions between the components of MLE and DEPs were further assessed using molecular docking.

**Results:**

After 8 weeks of MLE intervention, the clinical indications of T2DM such as body weight, food and water intake of rats were improved to a certain extent, while insulin sensitivity was increased and glycemic control was improved. Serum lipid profiles were significantly reduced, and the skeletal muscle fiber gap and atrophy were alleviated. Proteomic analysis of skeletal muscle showed that MLE treatment reversed 19 DEPs in T2DM rats, regulated cholesterol metabolism, fat digestion and absorption, vitamin digestion and absorption and ferroptosis signaling pathways. Key differential proteins Apolipoprotein A-1 (ApoA1) and ApoA4 were successfully validated by western blot and exhibited strong binding activity to the MLE’s ingredients.

**Conclusions:**

This study first provided skeletal muscle proteomic changes in T2DM rats before and after MLE treatment, which may help us understand the molecular mechanisms, and provide a foundation for developing potential therapeutic targets of anti-T2DM of MLE.

**Graphical Abstract:**

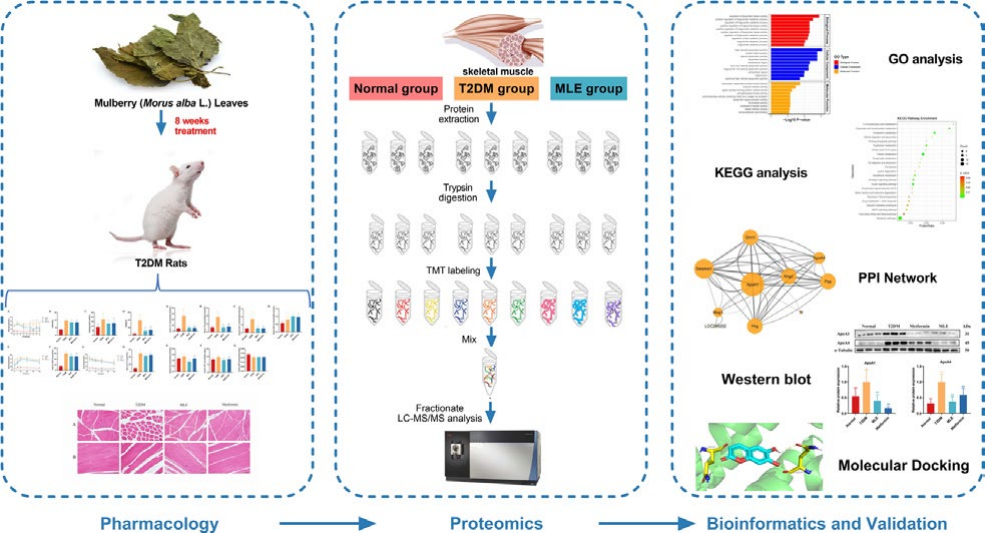

**Supplementary Information:**

The online version contains supplementary material available at 10.1186/s12906-023-04140-3.

## Introduction

Diabetes mellitus (DM) is a chronic disease with disorders of sugar, lipid and protein metabolism due to relative or absolute deficiency of insulin secretion [1]. As modern living standards improve and the population ages, the incidence of DM is increasing and has become one of the most threatening metabolic diseases [2]. Type 2 diabetes mellitus (T2DM), characterized by chronic hyperglycemia and insulin resistance (IR), accounts for over 90% of diabetes cases [3, 4]. Long-term hyperglycemia can cause damage to macrovascular and microvascular and lead to various complications such as stroke, kidney disease, cardiovascular disease, diabetic foot, and infections, which seriously affect the quality of life and long-term survival of patients [5, 6]. Therefore, it is of great social importance and economic value to elucidate the pathogenesis of T2DM and to find therapeutic drugs with definite efficacy and clear mechanism.

Skeletal muscle is an organ with high mitochondrial content, accounting for about 40% of body weight [7]. As an important peripheral tissue responsible for the uptake and processing of glucose, it is one of the main affected organs in DM [8]. DM is accompanied by severe hyperglycemia and abnormal fat and protein metabolism, leading to skeletal muscle atrophy, ectopic lipid deposition and mitochondrial dysfunction, which further aggravate T2DM and cause complications such as sarcopenia and fatigue [9–11]. Therefore, using skeletal muscle as an entry point to explore its changes in T2DM is important to explore the pathogenesis and therapeutic targets of T2DM.

In China, mulberry (*Morus alba* L.) leaf has been used as a traditional medicine or functional food for the treatment of T2DM, with a history dating back to the sixteenth century, recorded in Li Shizhen's Compendium of Materia Medica (“Ben Cao Gang Mu” in Chinese) [12]. Modern pharmacological studies have confirmed that the aqueous [13] and alcoholic [14] extracts of mulberry leaf and their polysaccharides [15], flavonoids [16, 17], alkaloids [18], phenolic acids [19], and other components are effective in preventing and treating T2DM [20]. 1-deoxynojirimycin (1-DNJ), a piperidine alkaloid isolated from mulberry leaf, is an α-glucosidase inhibitor and considered as one of the effective chemicals for anti-hyperglycemic [21, 22]. Our previous study showed that mulberry leaf significantly reduced blood glucose and lipid levels and improved glucose tolerance in T2DM rats, suggesting its anti-diabetic effect. Its effect may be related to inhibiting α-glucosidase [23], regulating toll-like receptors and insulin signaling pathways [24], activating brown adipose tissue and inducing browning of white adipose tissue [25]. However, its mechanisms of action have not been totally clarified.

The complex pathogenesis of T2DM coincides with the "multi-pathway, multi-component and multi-target" characteristics of TCM. Both have networked interactions between various components of different signaling pathways. With such a complex background, traditional research methods focusing on a single pathway are too limited, while omics technologies focusing on systemic and holistic features to reveal life processes meet the needs of the research. Proteomics focuses on all proteins in tissue samples to study protein expression, modification, function, and interactions in the body at a holistic level so as to better understand cellular life activity patterns and disease pathogenesis [26, 27]. Alterations in protein expression levels or post-translational modifications are directly related to disease occurrence and drug stress, therefore, proteomic analysis can visualize the changes in the internal tissues and organs of the organism and has unique advantages in exploring the mechanism of disease and drug treatment. In this study, skeletal muscle was taken as the research object, and proteomics techniques were used to explore the pathological mechanism of T2DM and the mechanism of mulberry leaf in the treatment of T2DM at the protein molecular level, which provides a basis for the development of potential therapeutic targets of mulberry leaf against T2DM.

## Materials and methods

### Preparation and identification of mulberry leaf extract

Mulberry leaf (Beijing Tong Ren Tang Co. Ltd., Beijing, China) (15 kg) was extracted with 10 times the mass of deionized water at 85 ℃ for 10 h and refluxed for 90 min. Then, the extracts were filtered and concentrated to 3.75 g crude drug /mL, sub-packed and stored at -80 ℃ for standby application [24].

Mulberry leaf aqueous extract was centrifuged at 12,000 r/min for 10 min and the supernatant was filtered through a 0.22 μm filter membrane as the test solution. Chromatographic separation was performed with a Vanquish UHPLC system (Thermo Fisher Scientific, Germany) with a Zorbax Eclipse C18 (2.1 mm × 100 mm, 1.8 μm, Agilent Technologies, USA) column. The mobile phases were 0.1% formic acid aqueous solution (A) and acetonitrile (B) with gradient elution, 0–2 min 5% B, 2.1–6 min 5–30% B, 6.1–7 min 30% B, 7.1–12 min 30–78% B, 12.1–14 min 78–95% B, 17.1–20 min 95% B, 20.1–21 min 95–5% B, and 21.1–25 min 5%B at a flow rate of 0.3 mL/min, temperature of 30 °C, and the injection volume was 2 μL. Mass spectrometry was analyzed by Q-Exactive HF high-resolution mass spectrometry (Thermo Fisher Scientific, Germany). The electrospray ionization source (ESI) was worked in positive and negative ion modes, respectively. The source parameters are as follows: electrospray voltage: 3.5 kV; heater temperature: 325 ℃; capillary temperature: 330 ℃; sheath gas flow rate: 45 arb; auxiliary gas flow rate: 15 arb; sweep gas flow rate: 1 arb; and S-Lens RF Level: 55%. MS data were acquired in full scan (m/z 100 ~ 1500) and 10 scan data-dependent MS/MS scan (dd-MS2, TopN method) mode. The MS1 and MS2 resolutions were set to 120,000 and 60,000, respectively, with higher-energy collisional dissociations (HCD). Compound Discoverer 3.3 was used for peak alignment, retention time correction and peak area extraction. Compounds were initially identified by searching mzCloud (Thermo Fisher Scientific), mzVault, and local databases.

### Animals and treatments

Wistar rats (SPF grade, male, 200–220 g) were purchased from Beijing Vital River Laboratory Animal Technology Co., Ltd. with experimental animal production license number SCXK (Jing) 2021–0006. All rats were housed in a barrier environment at the Beijing University of Chinese Medicine, and the experimental animal use license number is SYXK (Jing) 2020–0033. Animals were placed in an environment with a temperature of 22–24 ℃, relative humidity of 50–70%, and a light–dark cycle of 12 h. The animal study was reviewed and approved by the Medical and Experimental Animal Ethics Committee of Beijing University of Chinese Medicine (No. BUCM-4–2,019,070,305-3098).

All rats adapted to the environment with standard ordinary feed for one week, and there was no significant difference in body weight, food and water intake and fasting blood glucose (FBG) of all rats before the experiment. Then, rats were randomly assigned into two groups based on similar mean body weights: a normal group (*n* = 9), which was fed with the ordinary diet composed of 20.0% protein, 4.0% fat, 5.0% fiber, 8.0% ash, 1.0–1.8% calcium, and 0.6–1.2% phosphorus (1032, Beijing Hua Fukang Biological Technology Co., Ltd., Beijing, China); and a high-fat diet (HFD) group, which was fed with high-sugar and high-fat diet containing 63.6% (w/w) basic feed, 15% (w/w) lard, 20% (w/w) sucrose, 1.2% (w/w) cholesterol, and 0.2% (w/w) sodium cholate (H10601, Beijing Hua Fukang Biological Technology Co., Ltd., Beijing, China). After 4 weeks of feeding, the rats were fasted for 12 h with free access to water. The rats in the HFD group were injected intraperitoneally with 1% streptozotocin (STZ, Sigma, USA) dissolved in sodium citrate buffer (0.1 mmol/L, pH = 4.2–4.5, 4 ℃) at a dose of 35 mg/kg, while the normal rats were injected with an equal volume of sodium citrate buffer intraperitoneally. Blood was collected from the tail vein of rats on the 7th and 8th day after injection to measure FBG. And rats with FBG ≥ 11.1 mmol/L were randomly divided into three groups according to the body weight and blood glucose level as follows (*n* = 9/group): T2DM group, mulberry leaf extract (MLE, 4.0 g crude drug /kg) treatment group and metformin (200 mg/kg) treatment group. The optimal administration dosage of MLE was selected according to the previous study [25]. The animals were treated orally at a dose of 10 mL/kg once daily for 8 weeks. Additionally, rats in the normal and T2DM groups were gavaged with an equal amount of distilled water. During the experiment, the rats' water consumption, food intake and body weight were measured weekly, FBG was measured by collecting blood from tail veins after overnight fasting once every two weeks for the first 4 weeks and then weekly for the next 4 weeks.

### Oral glucose tolerance test (OGTT) and insulin tolerance test (ITT)

OGTT was performed in the 7th week of treatment, all rats fasted for 12 h with free access to water and then gavaged with 50% glucose solution (2 g/kg). For the ITT, rats were intraperitoneally injected with insulin (0.75 U/kg) after 4 h fasting during the 8th week of treatment. Afterward, the blood glucose was measured at 0, 15, 30, 60 and 120 min, and the area under the curve (AUC) was calculated as follows:

$$\mathrm{AUC}(\mathrm{mmol}/\mathrm{hL})=(\mathrm{BG}0+\mathrm{BG}15)\times 1/4\times 1/2+(\mathrm{BG}15+\mathrm{BG}30)\times 1/4\times 1/2+(\mathrm{BG}30+\mathrm{BG}60)\times 1/2\times 1/2+(\mathrm{BG}60+\mathrm{BG}120)\times 1/2$$

BG is the blood glucose value at each time point.

### Biochemical analysis

After the final administration, the rats were anesthetized with pentobarbital (40 mg/kg, *ip.*) and serum was obtained by centrifugation of blood collected from the abdominal aorta. Then the rat euthanized by cervical dislocation. The glycated hemoglobin A1c (HbA1c) and insulin levels were measured in whole blood samples using the HbA1c assay kit (Sekisui Medical Technology (China) Ltd., Beijing, China) and [^125^I] Insulin Radioimmunoassay Kit (Beijing Northern Biological Technology Research Institute, China) respectively. Serum levels of total cholesterol (TC), triglyceride (TG), low-density lipoprotein cholesterol (LDL-C) and high-density lipoprotein cholesterol (HDL-C) were measured by biochemical assay kits (Nanjing Jiancheng Bioengineering Institute, Nanjing, China). Serum free fatty acids (FFA), leptin (LEP) and adiponectin (ADP) were detected by ELISA kits (Kete Biotechnology Co., Ltd., Jiangsu, China). The Homeostasis Model of Insulin Resistance (HOMA-IR) index as a method of assessing insulin resistance was calculated as the following formula:

$$\mathrm{HOMA}-\mathrm{IR }= (\mathrm{fasting\ insulin }\times \mathrm{ fasting\ glucose})/22.5.$$

### Histopathological examination

Skeletal muscle tissues (gluteus maximus) were fixed in 10% neutral-buffered formalin for 24 h. The tissue was cut into 5-μm-thick sections after the steps of dehydration, transparency, wax immersion and embedding. The pathological alterations of skeletal muscle were observed through histopathological examination (HE) staining. Tissue damage was scored as described below. Briefly, scores were defined as (0)-none/none, (1) slight atrophy of skeletal muscle fibers, (2) mild atrophy of skeletal muscle fibers, (3) moderate atrophy of skeletal muscle fibers, and (4) severe atrophy of skeletal muscle fibers. Data were collected from 3 mice in each group.

### TMT-based quantitative proteomics analysis

#### Protein extraction, digestion and TMT labeling

The skeletal muscle samples of rats in the normal, T2DM and MLE groups were powdered with liquid nitrogen and extracted with lysis buffer containing 7 M urea, 2 M thiourea, 0.1% CHAPS and 1% protease inhibitor cocktail at 1:10 (W/V) ratio. After a 30-min incubation on ice, samples were centrifuged at 13,000 rpm for 15 min at 4 °C and the protein supernatant was collected. BCA assay (Biyuntian Biotechnology Co., Ltd., Shanghai, China) and SDS-PAGE were used to detect protein concentration and purity, respectively. To minimize individual rat differences, every three independent skeletal muscles within one group were mixed to extract proteins, and three samples were prepared for each group.

The samples were digested using the filter-aided sample preparation (FASP) method [28, 29]. In brief, protein samples were reduced through 25 mM dithiothreitol (DTT) (final concentration) at 37 ℃ for 1 h and then alkylated with 50 mM alkylation (IAA) (final concentration) in the dark at room temperature for 30 min, and subsequently digested with trypsin (Promega, USA) at a mass ratio of 1:50 (trypsin: protein) overnight at 37 ℃. The peptides digested by trypsin were dissolved in 0.1 M TEAB and labeled using the tandem mass tag (TMT) kit (Thermo Fisher Scientific, USA) according to the manufacturer’s protocol. The labeled peptide mixtures were desalted using solid-phase extraction columns (Oasis HLB, Waters, Milford, MA, USA) and then, vacuum dried.

#### Peptide fractionation with reverse phase (RP) chromatography and LC–MS/MS analysis

TMT-labeled peptides were fractionated by RP chromatography using High-Performance Liquid Chromatography Systems L-3000 (RIGOL TECHNOLOGIES, INC Beijing, China). The peptide mixtures were dissolved in buffer A (98% ddH_2_O, 2% acetonitrile, adjusted pH to 10.0 using NH_3_·H_2_O), and loaded onto an XBridge® peptide BEH C18 Column (130 Å, 3.5 μm, 4.6 mm × 150 mm) (Waters Corporation, USA). The peptides were eluted at a flow rate of 1 mL/min with a gradient of 5.0% buffer B (98% acetonitrile, 2% ddH_2_O, pH 10.0) for 10.00 min, 5.0–8.5% buffer B for 10.00–13.40 min, 8.5–20.5% buffer B for 13.40–31.70 min, 20.5–31.0% buffer B for 31.70–41.00 min, 31.0–90.0% buffer B for 41.00–46.00 min, 90.0–95.0% buffer B for 46.00–47.00 min, 95.0–5.0% buffer B for 47.00–48.00 min, 5.0% buffer B for 48.00–51.00 min. Fractions were collected at 1 min intervals, combined into 10 pooled fractions in the order 1, 11, 21, 31; 2, 12, 22, 32; 3, 13, 23, 33, etc., and vacuum freeze-dried for liquid chromatography with tandem mass spectrometry (LC–MS/MS) analysis. Each fraction was resuspended in buffer A (ddH_2_O with 0.1% formic acid) and loaded into an UltiMate 3000 UHPLC system (Thermo Fisher Scientific, USA) with a self-filling C18 reversed-phase analytical column (150 μm × 150 mm, 1.9 μm). Buffer A and buffer B (80% acetonitrile and 0.1% formic acid) were used as mobile phases. The flow rate was 0.6 μL/min with the following liquid gradient: 0–10.00 min, 6% buffer B; 10.00–15.00 min, 6–10% buffer B; 15.00–80.00 min, 10–30% buffer B; 80.00–90.00 min, 30–40% buffer B; 90.00–90.10 min, 40–95% buffer B; 90.10–95.00 min, 95% buffer B; 95.00–95.10 min, 95–6% buffer B; 95.10–100.00 min, 6% buffer B. The separated peptides were analyzed using an Orbitrap Q-Exactive HF-X mass spectrometer (Thermo Fisher Scientific, USA). The scanning range of the primary mass spectrum was set to 350–1500 m/z, the scanning resolution was set to 120,000 dpi, the automatic gain control (AGC) target was set to 3e6, and the maximum injection time was set to 50 ms. After the first stage scan, select the top 25 peptide precursor ions with the highest signal intensity to enter the high-energy collisional dissociation cell sequentially with a fragmentation energy of 32%, followed by secondary mass spectrometry. The starting m/z of the secondary mass spectra is fixed at 100, with the resolution set to 30,000 dpi. Charge state screening was enabled to include precursors from 2 to 7 charge states, and the dynamic exclusion time was set as 30 s.

#### Database search and bioinformatics analysis

After the MS analysis, all the raw MS/MS data were submitted to the MASCOT (Matrix Science, London, UK) and compared against the UniProt rat protein database. The following parameters were set: all tryptic specificity was required; max missed cleavages was two; carbamidomethyl (C) was set as the fixed modifications; the oxidation (methionine, M), and Acetyl (N-terminal) were set as the variable modifications; peptide mass tolerances were set at ± 10 ppm; fragment mass tolerances were set at 0.05 Da. The false discovery rate (FDR) was set as ≤ 0.01. The protein quantification was calculated as the median of only unique peptides of the protein. All peptide ratios were normalized by peptide median. Protein abundance was checked for differences between any two samples using Student's t-test. The upregulation threshold was set to a ratio > 1.2 and *P* < 0.05 for the comparison group, and the downregulation was set to a ratio < 0.83 and *P* < 0.05 for the comparison group to identify differentially expressed proteins (DEPs) in this study [30].

The DEPs were analyzed with Gene Ontology (GO) including Biological Process (BP), Cellular Component (CC), and Molecular Function (MF) using OmicsBox 1.2.4/Blast2Go. Pathway enrichment analysis of DEPs was performed using the DAVID. Functional categories and pathways with *P* < 0.05 and FDR < 0.05 were considered significant. Protein–protein interactions (PPI) were analyzed on the STRING database (http://string-db.org) with a required minimum interaction score of 0.4. and visualized by Cytoscape 3.7.2 software.

### Western blotting validation

Western blot was carried out to validate the TMT-based proteomics analysis results. Skeletal muscle total protein was extracted with RIPA lysis buffer containing protease and phosphatase inhibitors, and the concentrations were determined using a BCA protein assay kit (P0010S, Beyotime, China). The total protein (10 μg) from each sample was separated by SDS-PAGE gel electrophoresis and then electrotransferred onto PVDF membranes. After 1.5 h of blocking in the blocking solution, the PVDF membrane containing the proteins was incubated in the desired primary antibody, including Anti-ApoA1 antibody (1:1000, ab227455, Abcam, UK), Anti-ApoA4 antibody (1:10,000, 5700S, Cell Signaling Technology, USA) and Anti-alpha Tubulin antibody (1:5000, ab7291, Abcam, UK) overnight at 4 ℃. After incubating with HRP-conjugated Affinipure Goat Anti-Mouse IgG (H + L) (1:5000, SA00001-1, Proteintech, USA) or HRP-conjugated Affinipure Goat Anti-Rabbit IgG (H + L) (1:5000, SA00001-2, Proteintech, USA) for 1 h, PVDF membrane was washed with TBST, incubated with an electro-chemiluminescence (ECL) developing solution, and exposed and photographed. Using ImageJ, target protein bands are measured and their relative levels of expression are quantified by the ratio of the corresponding protein to α-tubulin.

### Molecular docking

The above-obtained DEPs ApoA1 and ApoA4, and the active compounds of MLE were selected for the molecular docking analysis. The compounds' structures were obtained from the PubChem database (https://pubchem.ncbi.nlm.nih.gov/) [31], and the ApoA1 (4UYF) [32] and ApoA4 (3S84) [33] PBD format files were downloaded from the RCSB Protein Data Bank (http://www.rcsb.org/) [34]. The proteins and ligands were further modified using AutoDock 4.2 by removing water molecules and adding polar hydrogen atoms and Gasteiger charges. Molecular docking was then performed using AutoDock 4.2 and visualized using Pymol software.

### Statistical analysis

GraphPad Prism 8 and SPSS 22.0 were used to statistically analyze and present the experimental data as mean ± standard deviation (SD). When each group's data had a normal distribution and equal variances, one-way ANOVA was performed, and the LSD method was used to compare the groups. A nonparametric test was performed when the data did not follow a normal distribution or the variance was not homogeneous. *P*-value < 0.05 indicated that the difference was statistically significant.

## Results

### Identification of chemical compounds from MLE

**Fig. 1:**
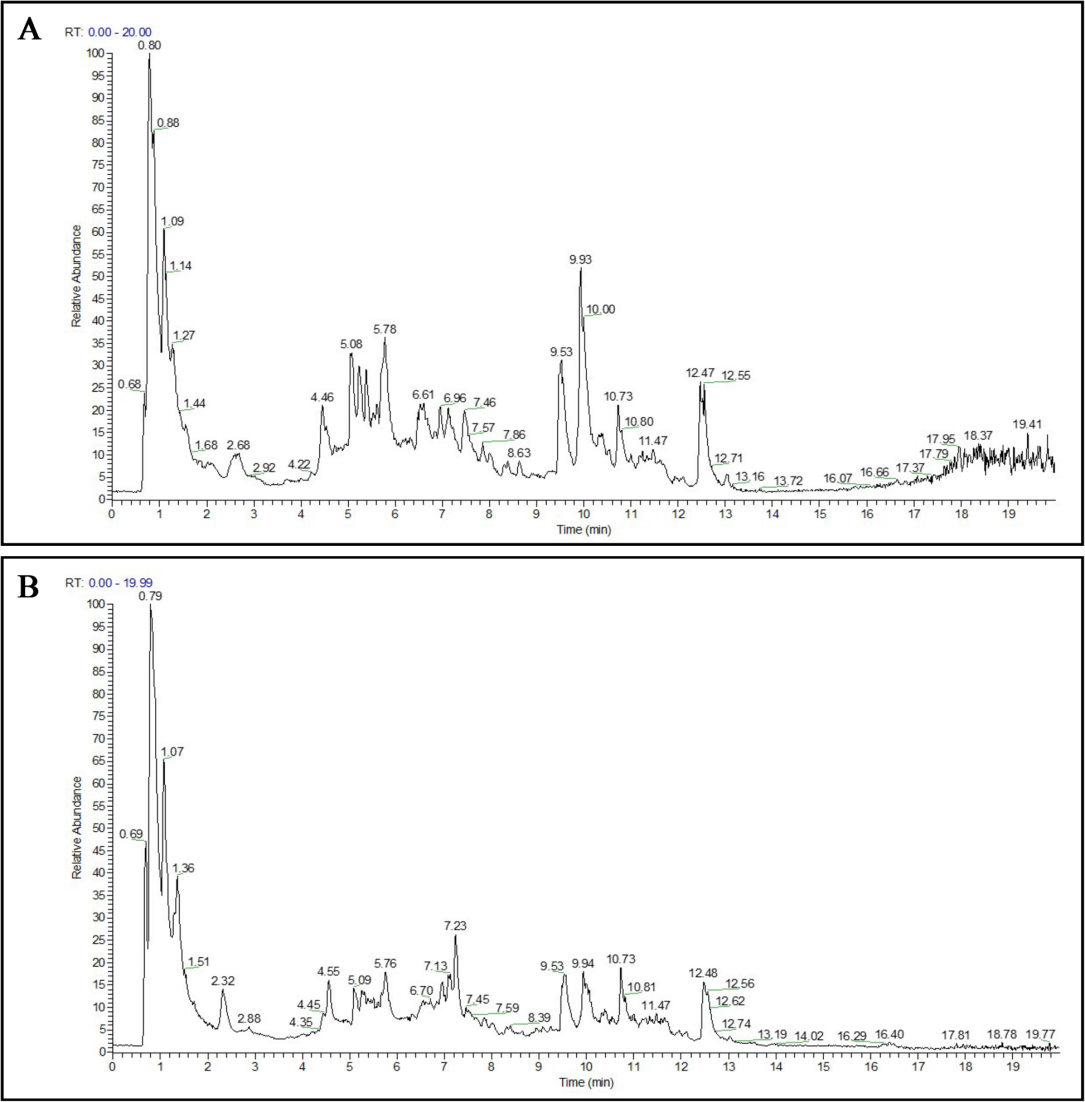
Total ion chromatogram of mulberry leaf extract in negative-ion mode (**A**) and positive-ion mode (**B**)

As shown in Fig. 1A, B, a total of 31 compounds, namely one benzene (Gallic acid), three Carboxylic acids (Betaine, DL-Stachydrine and L(-)-Pipecolinic acid), two Cinnamic acids (2-Hydroxycinnamic acid and Caffeic acid), five Coumarins (7-Hydroxycoumarine, Esculin, Esculetin, Scopolin and Scopoletin), one Fatty Acyls (Quercetin-3-O-β-D-glucose-7-O-β-D-gentiobioside), 11 Flavonoids (Quercetin-3,4'-O-di-beta-glucoside, Isoquercitrin, Rutin, Quercetin-3β-D-glucoside, Luteolin 7-glucuronide, Kaempferol-3-O-rutinoside, Kaempferol, Kaempferol-7-O-glucoside, Luteolin, Quercetin and Kuwanon C), one Isoflavonoids(Formononetin), four Organooxygen compounds (3,4-Dihydroxybenzaldehyde, Chlorogenic acid, Salidroside and Isochlorogenic acid B), one Piperidines (1-Deoxynojirimycin), two Stilbenes (Mulberroside A and (E)-Resveratroloside) were identified by the retention time and the MS/MS spectra data in negative and positive ion modes. Retention time, experimental and calculated molecular weight, molecular formulas, errors in parts per million (ppm), major MS/MS fragments, and other information are displayed in Supplementary Table S1. For all identified compounds, the mass error was below 5 ppm.

### Effects of MLE treatment on body weight, and food and water intake

**Fig. 2:**
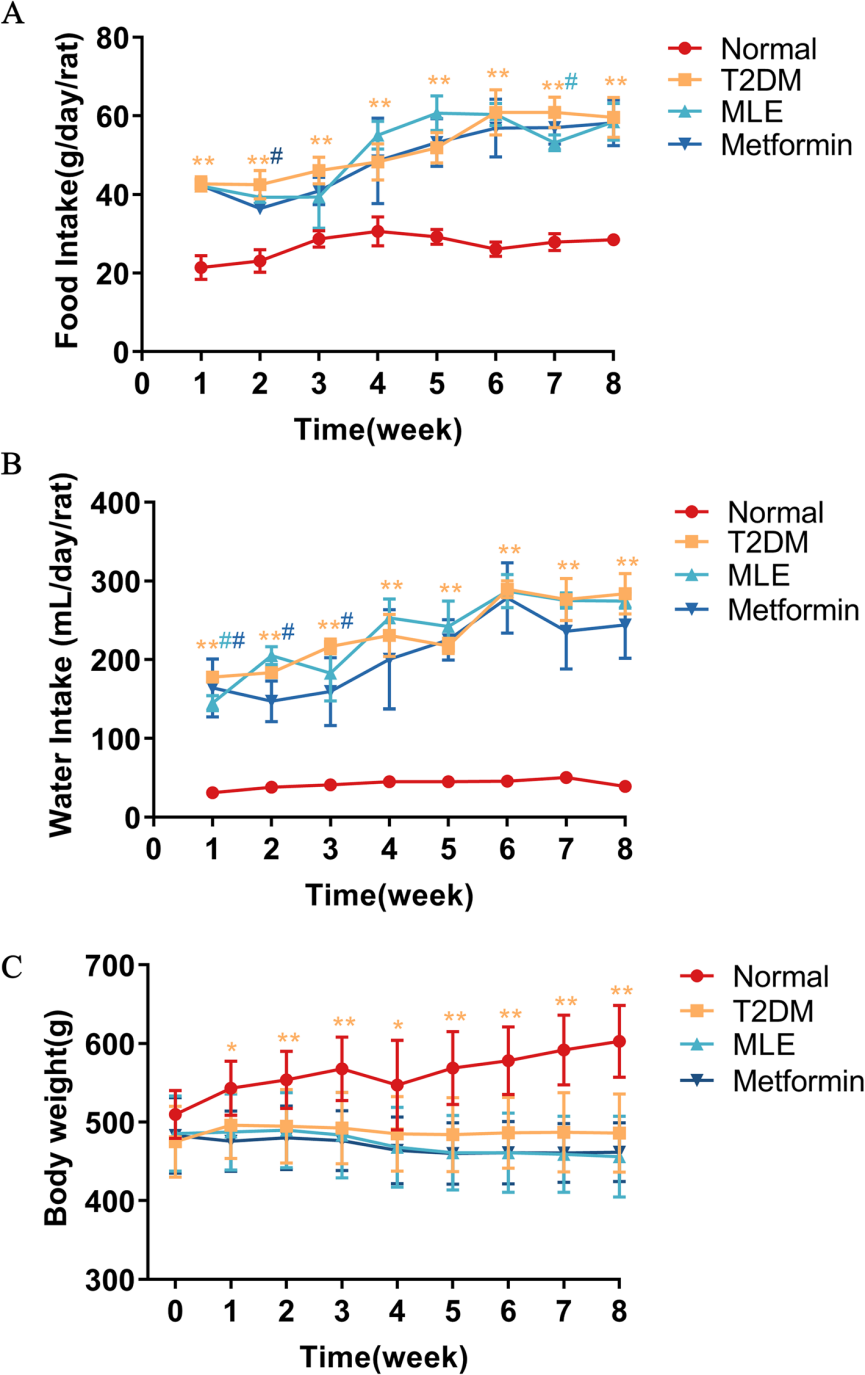
Effects of MLE on food intake (**A**), water intake (**B**) and body weight (**C**) in T2DM rats. Data were shown as mean ± SD (*n* = 9). *P < 0.05, **P < 0.01 vs. normal group; #*P* < 0.05, ##*P* < 0.01 vs. T2DM group. *T2DM* Type 2 diabetes mellitus, *MLE* Mulberry leaf extract. Week 0 = week of treatment, the first day of week 0 is the day of treatment. Note that the color of the * and # indicating significance match the color of the respective group

Compared with normal rats, T2DM rats showed a significant increase in food and water intake (*P* < 0.01) (Fig. 2A, B) while body weight was significantly decreased (*P* < 0.05 or *P* < 0.01) (Fig. 2C). Compared with the T2DM group, the rats in the MLE group had significantly lower water intake at week 1 (*P* < 0.05) and lower food intake at week 7 (*P* < 0.05). The water intake of rats in the metformin group was significantly lower (*P* < 0.05) in weeks 1, 2, and 3, and the food intake was significantly lower (*P* < 0.05) in week 2. However, no significant change was found in the body weight of rats in the MLE and metformin groups compared with the T2DM group.

### Effects of MLE treatment on hyperglycemia

**Fig. 3:**
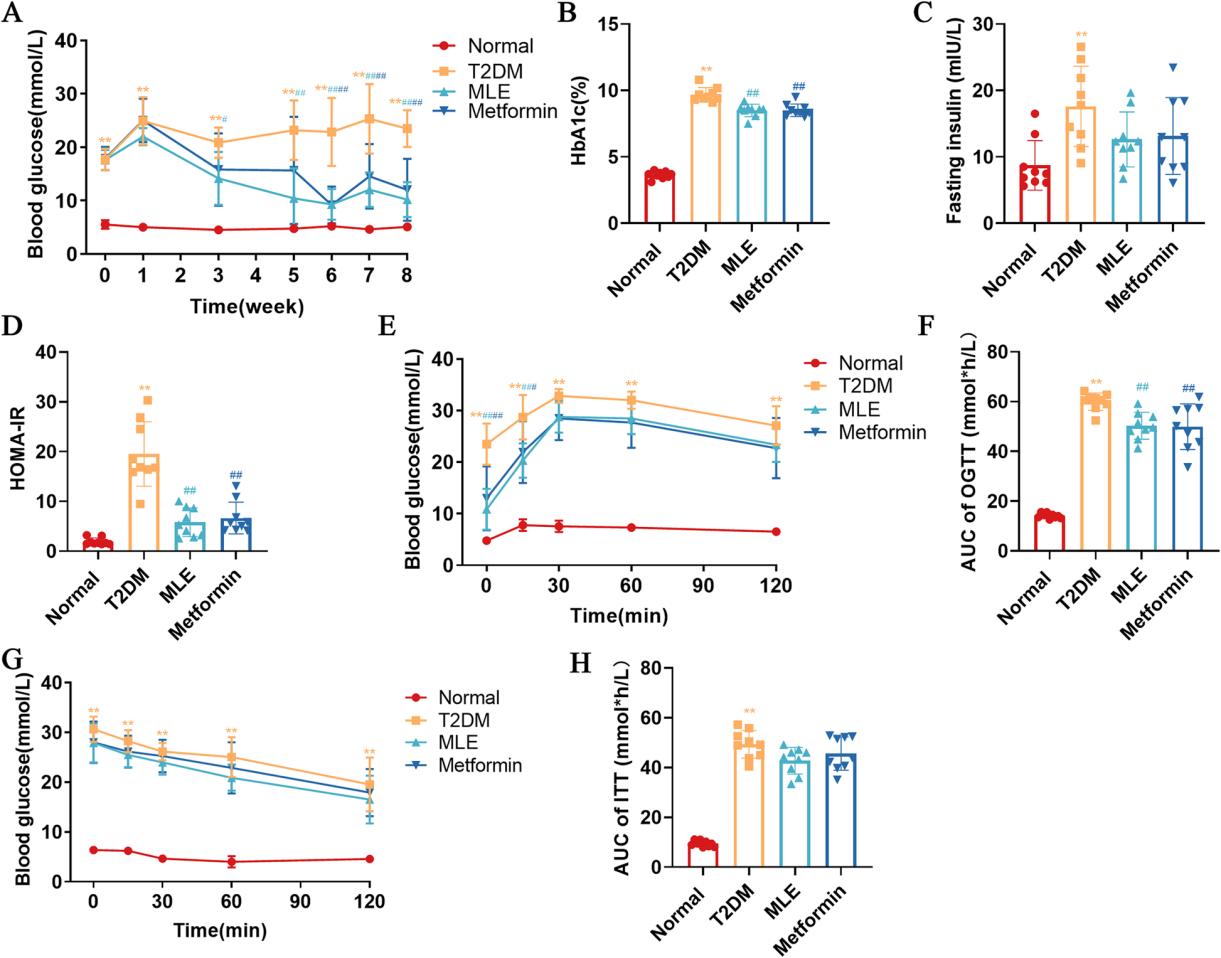
Effects of MLE treatment on hyperglycemia. **A** Fasting blood glucose. **B** Glycated hemoglobin (HbA1c). **C** Fasting insulin. **D** HOMA-IR. **E** Oral glucose tolerance test (OGTT). **F** AUC of OGTT. **G** Insulin resistance test (ITT). **H** AUC of ITT. Data were shown as mean ± SD (*n* = 9). *P < 0.05, **P < 0.01 vs. normal group; #*P* < 0.05, ##*P* < 0.01 vs. T2DM group. *T2DM* Type 2 diabetes mellitus, *MLE* Mulberry leaf extract. Week 0 = week of treatment, the first day of week 0 is the day of treatment. Note that the color of the * and # indicating significance match the color of the respective group

The FBG was considerably higher in T2DM rats following the induction of experimental hyperglycemia compared to normal rats (*P* < 0.01) and stayed above 11.1 mmol/L throughout the research, demonstrating the viability and stability of the T2DM model. MLE significantly decreased the FBG of rats in weeks 3–8 compared to T2DM rats (*P* < 0.05 or *P* < 0.01), while metformin significantly decreased FBG in weeks 6–8 (*P* < 0.01) (Fig. 3A).

After 8 weeks of treatment, the HbA1c and fasting insulin levels of rats were measured, and the HOMA-IR index was estimated (Fig. 3B-D). The T2DM rats exhibited significantly higher levels of HbA1c, insulin, and HOMA-IR compared with the control group (*P* < 0.01). MLE and metformin significantly reduced HbA1c levels in rats compared with the model group (*P* < 0.01). Compared with the T2DM group, although the decrease in insulin levels was not significant in the MLE and metformin groups (*P* > 0.05), the HOMA-IR score was significantly lower (*P* < 0.01).

To evaluate the impact of MLE on systemic glucose homeostasis, OGTT and ITT were performed. (Fig. 3E-H). After gavage of 50% glucose in all groups, blood glucose increased significantly, reaching a peak at 15 min in the normal group and 30 min in the other groups, and then decreased. In the T2DM group, the blood glucose values were significantly higher than normal rats at all time points (*P* < 0.01), and the AUC was significantly higher (*P* < 0.01). Compared with the T2DM group, the blood glucose of rats in the MLE and metformin groups was significantly decreased at 0 and 15 min (*P* < 0.05 or *P* < 0.01), and the AUC index was significantly decreased (*P* < 0.01). The blood glucose of rats in each group was significantly lower after insulin injection. The blood glucose and AUC index of T2DM rats were significantly higher than those of normal rats (*P* < 0.01). While the MLE and metformin treated rats showed a trend for decreased blood glucose and AUC index in T2DM rats, no significant difference was observed.

### Effects of MLE treatment on blood lipid metabolism

**Fig. 4:**
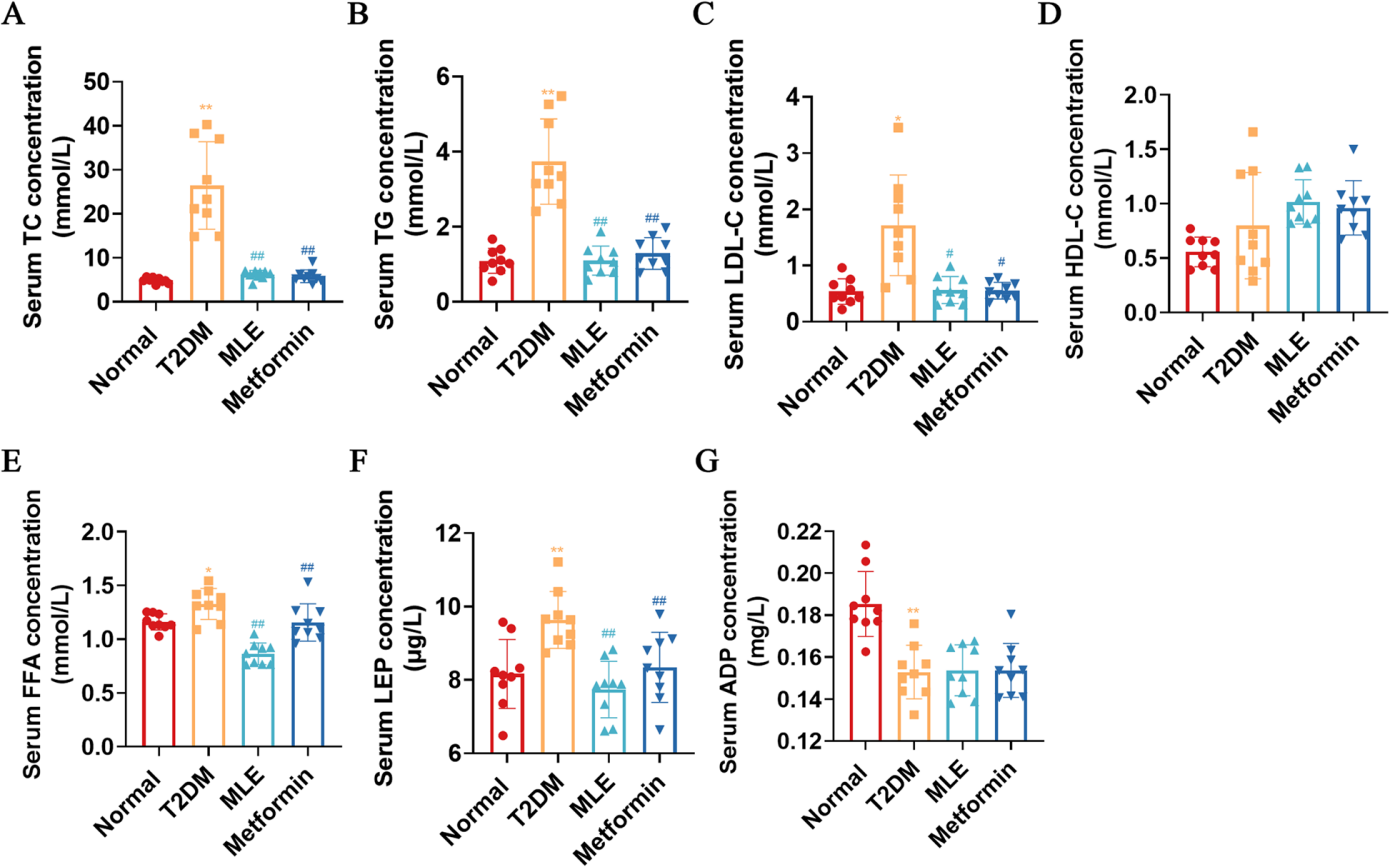
Effects of MLE treatment on Blood Lipid Metabolism. **A** Serum TC concentration. **B** Serum TG concentration. **C** Serum LDL-C concentration. **D** Serum HDL-C concentration. **E** Serum FFA concentration. **F** Serum LEP concentration. **G** Serum ADP concentration. Data were shown as mean ± SD (*n* = 9). *P < 0.05, **P < 0.01 vs. normal group; #*P* < 0.05, ##*P* < 0.01 vs. T2DM group. *T2DM* Type 2 diabetes mellitus, *MLE* Mulberry leaf extract

Subsequently, we measured the serum levels of TC, TG, LDL-C, HDL-C, FFA, LEP and ADP to examine the effects of MLE on lipid metabolism in T2DM rats (Fig. 4A-G). Compared with the normal group, TC, TG, LDL-C, FFA and LEP levels were significantly increased (*P* < 0.05 or *P* < 0.01) and ADP levels were significantly decreased (*P* < 0.01) in T2DM rats; MLE and metformin treatment significantly decreased serum TC, TG, LDL-C, FFA and LEP levels in rats (*P* < 0.05 or *P* < 0.01), but had no significant effect on ADP. No significant difference in HDL-C was noted among all groups.

### Effects of MLE treatment on skeletal muscle histomorphology

**Fig. 5:**
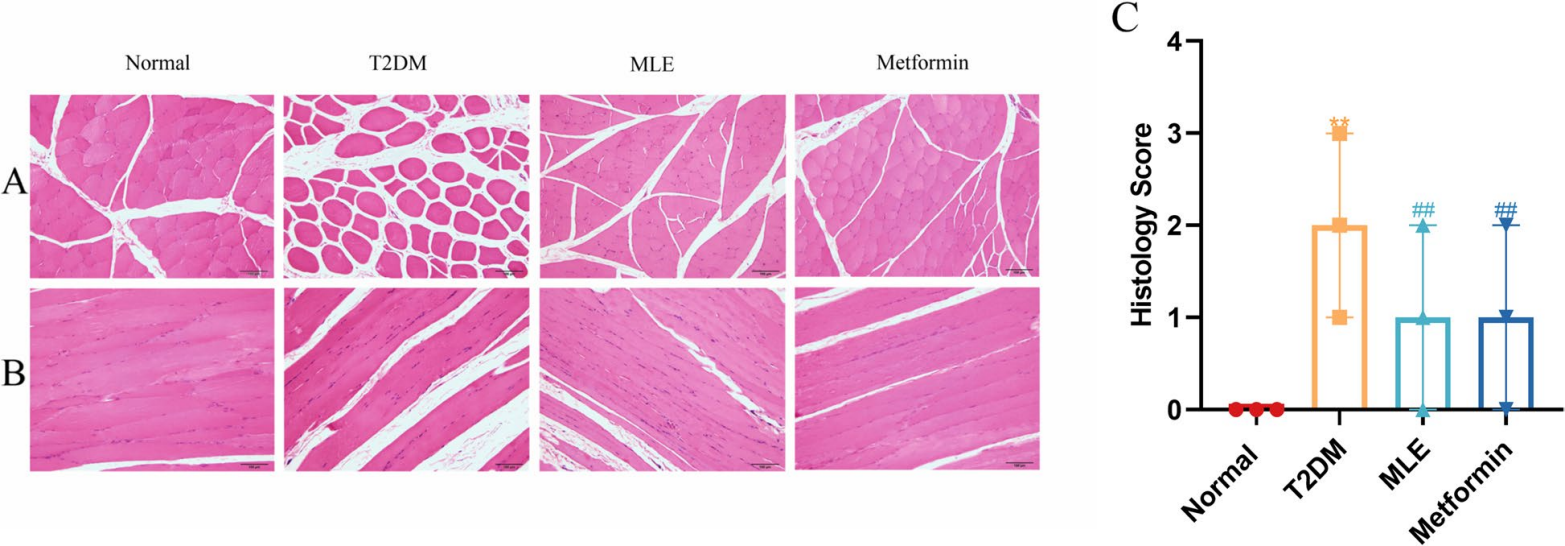
Effects of MLE on the histological features of skeletal muscle in rats. HE staining of skeletal muscle in (**A**) Transverse planes, (**B**) Longitudinal planes, (**C**) Histology score of skeletal muscle. × 20 magnification; scale bar = 100 μm; MLE, mulberry leaf extract

Skeletal muscle histomorphology was assessed by HE staining, and the results were shown in Fig. 5. Skeletal muscle fibers in normal rats were arranged regularly and closely with no evidence of histopathological changes. The skeletal muscle of T2DM rats was mildly atrophied, and the muscle fibers showed mild eosinophilic degeneration and widened gaps. After treatment with MLE and metformin, the skeletal muscle fibers of rats were neatly aligned, and the muscle fiber gap and atrophy were alleviated.

### Quantitative identification of differentially expressed proteins

**Fig. 6:**
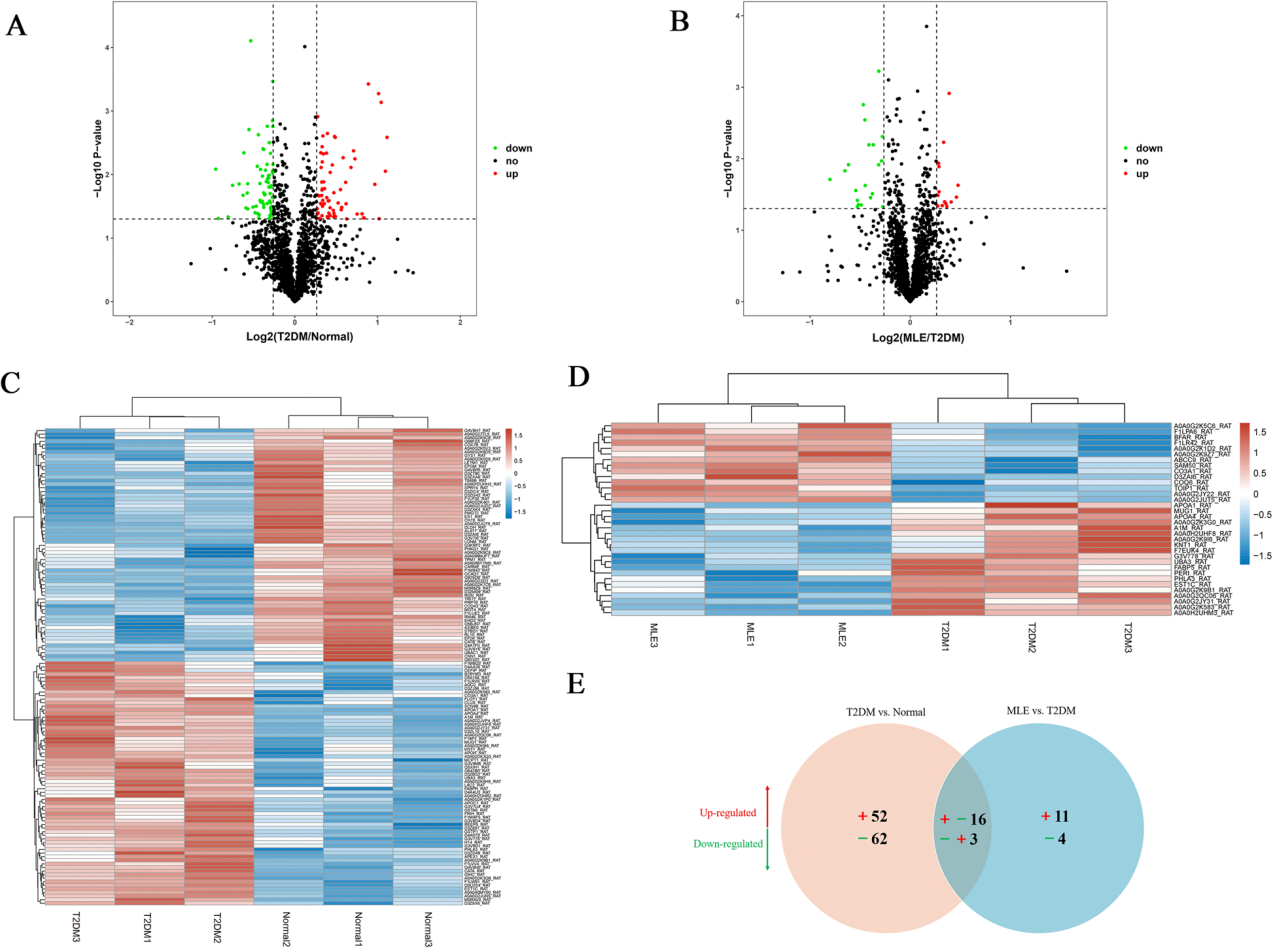
Differentially expressed proteins (DEPs) profiles by TMT-based proteomic analysis. The volcano plots of DEPs between T2DM versus normal groups (**A**) and MLE versus T2DM groups (**B**). The X-coordinate is the logarithmic value of the relative quantitative value of the protein after the Log2 conversion, and the Y-coordinate is the P-value after the -log10 conversion. The red dots indicate significantly up-regulated proteins, and the green dots indicate significantly down-regulated proteins. The heatmap of hierarchical clustering analysis on DEPs between T2DM versus normal groups (**C**) and MLE versus T2DM groups (**D**). Red blocks represent up-regulated proteins and blue blocks represent down-regulated proteins. **E** Venn diagram of the distribution and overlaps of DEPs between T2DM versus normal groups and MLE versus T2DM groups. *T2DM* Type 2 diabetes mellitus, *MLE* Mulberry leaf extract

To better decipher the molecular mechanisms and reveal skeletal muscle biomarkers of MLE treatment in T2DM rats, a TMT-labeled quantitative proteomic analysis was performed on skeletal muscle tissues. A total of 133 DEPs were identified between the T2DM and normal groups, of which 68 proteins were significantly up-regulated and 65 proteins were significantly down-regulated (Supplementary Table S2), whereas 34 DEPs were observed between the MLE and T2DM groups, including 14 significantly up-regulated proteins and 20 significantly down-regulated proteins (Supplementary Table S3). The protein expression patterns and DEPs were visualized via volcano plots in Fig. 6A, B. Hierarchical clustering showed significant differences in expression profiles among the three groups and good reproducibility among the three replicates of each group, indicating that the proteomic results were reliable (Fig. 6C, D). Furthermore, the Venn diagram showed the overlap of all DEPs between T2DM versus normal and MLE versus T2DM groups in Fig. 6E, MLE treatment significantly reversed the 16 up-regulated and 3 down-regulated proteins induced in the T2DM group (Supplementary Table S4), these proteins may be the key targets of MLE in the treatment of T2DM.

### Bioinformatics analysis of DEPs

**Fig. 7:**
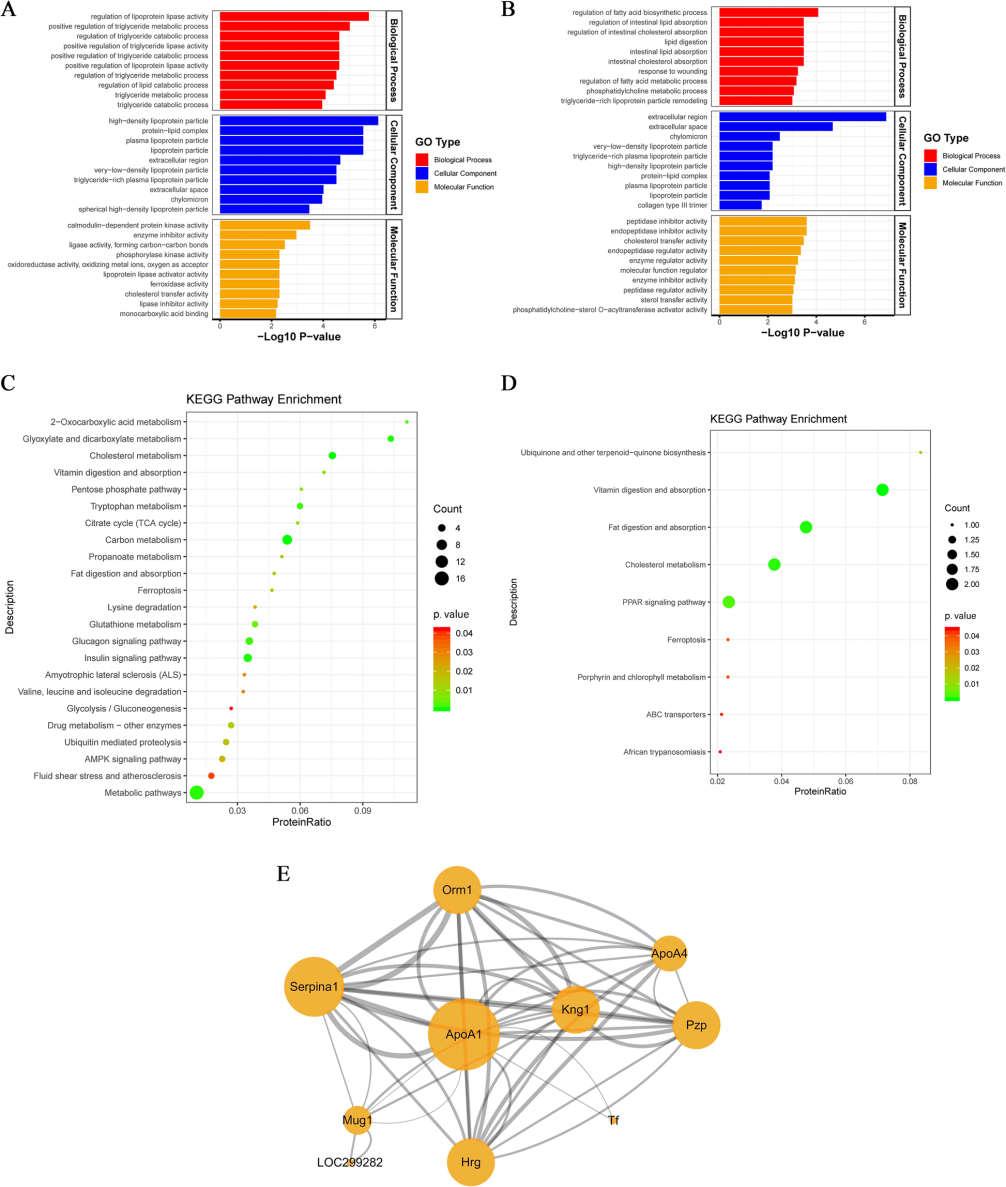
Bioinformatics Analysis of differentially expressed proteins (DEPs). GO annotation classification analysis of the DEPs between T2DM versus normal groups (**A**) and MLE versus T2DM groups (**B**). KEGG pathway enrichment analysis of the DEPs between T2DM versus normal groups (**C**) and MLE versus T2DM groups (**D**). **E** Protein–protein interaction network analysis of 19 overlapping proteins in the T2DM versus normal and MLE versus T2DM groups. The proteins are represented by a circle node. The node size represents the degree of the node; the larger the node size, the higher the degree. There is a positive correlation between the thickness of the edge and the binding score value between the proteins. *T2DM* Type 2 diabetes mellitus, *MLE* Mulberry leaf extract

As shown in Fig. 7A, B. GO enrichment analysis of DEPs between T2DM versus normal groups showed that the BP was significantly involved in the regulation of lipoprotein lipase activity, positive regulation of triglyceride metabolic process and regulation of triglyceride catabolic process and so forth; enriched CC annotations included high-density lipoprotein particle, protein-lipid complex and plasma lipoprotein particle, etc.; and the MF was mainly involved in calmodulin-dependent protein kinase activity, enzyme inhibitor activity, lipoprotein lipase activator activity, cholesterol transfer activity and lipase inhibitor activity, and so on. These results demonstrated that the pathogenesis of T2DM is mainly associated with abnormalities in lipid metabolism. DEPs between MLE versus T2DM groups participated in BP including regulation of fatty acid biosynthetic process, regulation of intestinal lipid absorption, regulation of intestinal cholesterol absorption and lipid digestion, etc.; CC included chylomicron, very-low-density, lipoprotein particle, triglyceride-rich plasma lipoprotein particle, high-density lipoprotein particle, protein-lipid complex and plasma lipoprotein particle, and so on; and the MF was related to peptidase inhibitor activity, endopeptidase inhibitor activity, cholesterol transfer activity, peptidase regulator activity and sterol transfer activity and so forth. These findings indicated that MLE treatment alleviated the impaired lipid metabolism to some extent in the experimental T2DM rats, which could be a potential mechanism for the treatment of T2DM.

In addition, the KEGG pathway enrichment analysis was performed. The DEPs between the T2DM and normal groups were significantly enriched in pathways involved in the insulin signaling pathway, glucagon signaling pathway, cholesterol metabolism, fat digestion and absorption, carbon metabolism, AMPK signaling pathway, glutathione metabolism, vitamin digestion and absorption, tryptophan metabolism, etc. (Fig. 7C**).** And the DEPs between the MLE and T2DM groups were highly enriched in pathways involved in PPAR signaling pathway, cholesterol metabolism, fat digestion and absorption, vitamin digestion and absorption, and so on (Fig. 7D**)**. According to the KEGG pathway enrichment analysis, MLE may exert a therapeutic effect on T2DM by interfering with lipid metabolism-related pathways, which was consistent with GO analysis.

PPI is essential for all biological processes since most proteins work through their interactions with other proteins. We further constructed the interaction network of 19 overlapping proteins in the T2DM versus normal and MLE versus T2DM groups using the STRING database and visualized it by Cytoscape (Fig. 7E**)**. There were 10 nodes and 25 edges in the PPI network (9 targets did not interact with each other, so they are not shown). Apolipoprotein A-1 (ApoA1), Alpha-1-antitrypsin (Serpina1), Pregnancy zone protein (Pzp), Alpha-1-acid glycoprotein 1 (Orm1), Histidine-rich glycoprotein (Hrg), Kininogen 1 (Kng1) and ApoA4 occupied the center of the PPI network and acted as hubs to interact with other DEPs. Among them, ApoA1 and ApoA4 participated together in very-low-density lipoprotein (VLDL) particle remodeling, high-density lipoprotein particle assembly, positive regulation of cholesterol esterification, reverse cholesterol transport, positive regulation of lipoprotein lipase activity and other biological processes related to lipid metabolism. Combined with the above analysis, we initially found that MLE reversed the upregulation of ApoA1 and ApoA4 expression in lipid metabolism in T2DM rats, so biological validation of ApoA1 and ApoA4 proteins will be performed subsequently.

### Validation of ApoA1 and ApoA4 proteins expression in rats

**Fig. 8:**
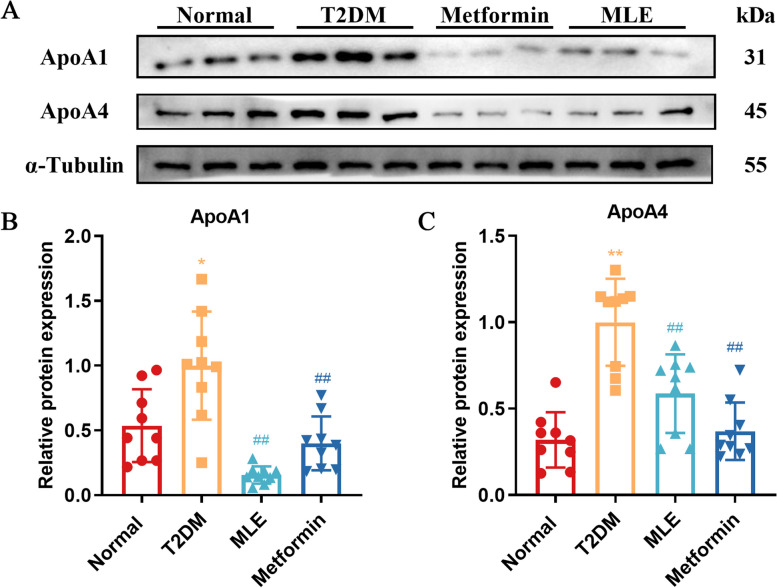
Validation of the expressions of ApoA1 and ApoA4 by Western blot analysis. **A** The western blotting assay was performed to measure the expression of ApoA1 and ApoA4 in the skeletal muscle of rats. Relative expression of ApoA1 (**B**) and ApoA4 (**C**) were quantified. Data were shown as mean ± SD (*n* = 9). *P < 0.05, **P < 0.01 vs. normal group; #*P* < 0.05, ##*P* < 0.01 vs. T2DM group. *T2DM* Type 2 diabetes mellitus, *MLE* Mulberry leaf extract. Full-length blots are presented in Supplementary Figure S1

As shown in Fig. 8, the protein expressions of ApoA1 and ApoA4 in the skeletal muscle of T2DM rats were increased compared with the normal rats (*P* < 0.05 or *P* < 0.01). Compared with the T2DM group, MLE and metformin treatment decreased the expressions of ApoA1 and ApoA4 significantly (*P* < 0.01). These results coincided with the quantitative trends in proteomics.

### Molecular docking

**Fig. 9:**
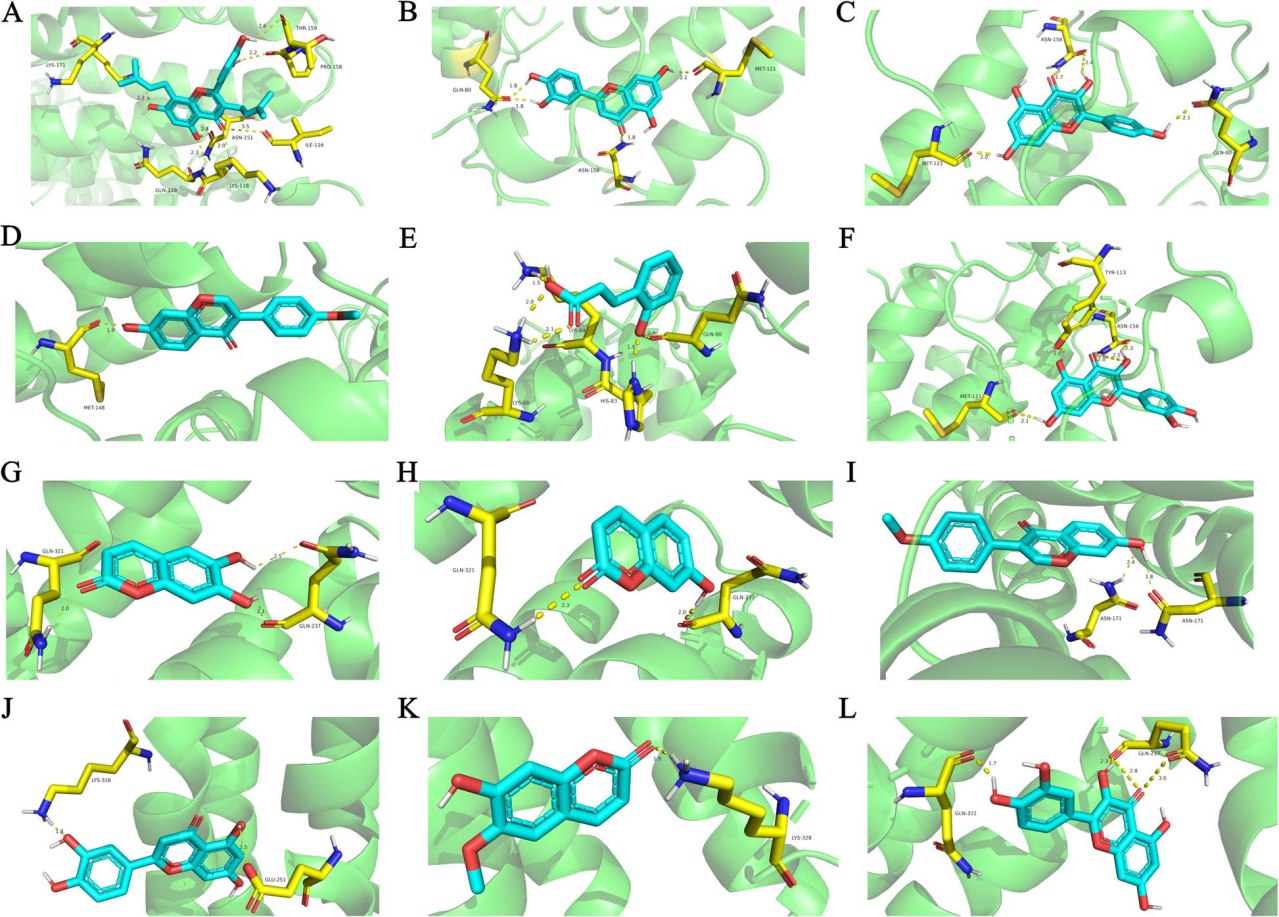
Components—Targets Molecular Docking. The molecular docking poses of Kuwanon C—ApoA1 (**A**), Luteolin—ApoA1 (**B**), Kaempferol—ApoA1 (**C**), Formononetin—ApoA1 (**D**), 2-Hydroxycinnamic acid—ApoA1 (**E**), Quercetin—ApoA1 (**F**), Esculetin—ApoA4 (**G**), 7-Hydroxycoumarine—ApoA4 (**H**), Formononetin—ApoA4 (**I**), Luteolin—ApoA4 (**J**), Scopoletin—ApoA4 (**K**), Quercetin—ApoA4 (**L**)

The interaction between the components and the targets was further assessed using molecular docking. The binding energy reveals the binding strength of the ligand and the receptor, the lower the binding energy, the more likely and stable the ligand-receptor interaction. The 31 compounds of MLE and two proteins ApoA1 and ApoA4 were used as ligands and receptors, respectively. The molecular docking results showed that most of the components had good binding activity for the targets with binding energies < 0 kcal/mol) [35]. The protein name, component name, PDB ID, and binding energy were shown in Supplementary Table S5. As shown in Fig. 9, Kuwanon C, Luteolin, Kaempferol, Formononetin, 2-Hydroxycinnamic acid and Quercetin showed the best binding activity to ApoA1 with the binding energy of -7.67, -7.3, -7.19, -6.82, -6.69 and -6.55 kcal/mol, respectively. Esculetin, 7-Hydroxycoumarine, Formononetin, Luteolin, Scopoletin and Quercetin showed the best binding activity to ApoA4 with a binding energy of -4.33, -4.21, -4.17, -3.9, -3.82 and -3.77 kcal/mol. Figure 9 presents the binding sites and binding interactions between the compounds and the targets.

## Discussion

T2DM is a metabolic disease caused by a complex interaction between genetics, hormone deficiency, metabolic and environmental factors (diet and lifestyle), characterized by hyperglycemia, hyperlipidemia, and IR. The pathogenesis of T2DM is complex, involving a large number of gene expressions, dysfunctions and alterations of multiple signaling pathways, which is compatible with the "multi-pathway, multi-component and multi-target" integrated effect of TCM. Chinese medicine can regulate the organism at the overall level and has significant advantages in the treatment of T2DM. Mulberry leaf, as a common medicinal and food homologous medicine for the treatment of T2DM in TCM, has a clear hypoglycemic effect with various active ingredients and pathways [36], but the specific mechanisms have not been fully elucidated. The present study aimed to provide a system-wide view of the therapeutic mechanism of MLE in T2DM treatment by applying TMT-labeled proteomics.

In the present study, the induction of T2DM was performed according to the method of Srinivasan et al. by HFD feeding combined with a low-dose STZ injection [37]. STZ has selective toxicity to islet β cells. In general, experimental animals can be fed a high-fat diet to make peripheral tissues insensitive to insulin [38] and injected with small amounts of STZ to destroy a part of the islet β-cell function [39]. In this experiment, the T2DM rat model showed significant hyperglycemia, hyperlipidemia, glucose intolerance and IR, accompanied by severe weight loss, which are similar to the pathophysiological features of human T2DM [40, 41]. The decrement in body weight observed in uncontrolled diabetic rats may be the result of protein loss due to the inability to use carbohydrates as an energy source, or to excessive diuresis featured in diabetic tables [42–44]. After 8 weeks of MLE intervention, the clinical indications of T2DM such as body weight, food and water intake of rats were improved to a certain extent, while insulin sensitivity was increased and glycemic control was improved. T2DM rats exhibit symptoms of hyperinsulinemia, which may be due to incomplete damage to the pancreas and insulin resistance in peripheral tissues. As MLE improved insulin resistance in rats, insulin levels decreased. This is consistent with the reported results in the literature [45, 46]. As expected, serum lipid profiles (including TC, TG, LDL-C, FFA and LEP) were significantly increased in T2DM rats due to excessive intake of HFD [47]. However, MLE treatment in these diabetic rats significantly reduced the serum lipid profile, suggesting that it has a good effect on regulating lipid metabolism. These findings were consistent with previous studies [25, 48].

To further reveal the mechanism of MLE for the treatment of T2DM, a TMT-based quantitative proteomics analysis was performed to screen DEPs, followed by bioinformatics analysis and validation. The common pathways involved in DEPs between the T2DM and normal groups and between the MLE and T2DM groups were cholesterol metabolism, fat digestion and absorption, vitamin digestion and absorption and ferroptosis. Ferroptosis is a form of non-apoptotic regulated cell death, characterized by intracellular iron overloaded and iron-dependent lipid peroxide accumulation [49, 50]. The common ferroptosis mechanisms result from cystine/glutamate antiporter (x_c_ −) system inhibition, glutathione (GSH) depletion, glutathione peroxidase-4 (GPX4) inactivation, lipid peroxidation, and iron dysregulation [50, 51]. Ferroptosis promotes the development of diabetes by participating in glucose-stimulated insulin secretion (GSIS) impairment and arsenic-induced pancreatic damage. In addition, iron and iron-sulfur (Fe-S) clusters interact, leading to mitochondrial iron accumulation, more reactive oxygen species (ROS) production, endoplasmic reticulum (ER) stress, failure of insulin biosynthesis, and ferroptosis in β-cell [52–54]. Among the DEPs between the T2DM and normal groups, 2 up-regulated proteins, Ceruloplasmin (Cp) and Ferritin heavy chain (Fth) were involved in ferroptosis. Cp is a ferroxidase that plays a crucial role in the oxidation of Fe^2+^ to Fe^3+^, thereby binding iron to transferrin and promoting iron mobilization in the body [55]. Cp synthesis and/or secretion can be altered by inflammation and insulin, and Cp levels are positively associated with hyperglycemia and IR [56, 57]. Fth is an important iron storage protein in the body [58]. Elevated Fth is associated with IR, while Fth is able to mediate apoptosis of islet cells through mitochondrial and death receptor apoptotic pathways. In addition, Fth can induce more ferritin formation through interaction with inflammatory factors, which exacerbates the incidence and development of T2DM [59]. MLE could reverse the increased Cp protein level and inhibit ferroptosis in T2DM rats, which may be one of the mechanisms of its IR improvement and hypoglycemic effect.

Lipids are involved in energy metabolism, transport of fat-soluble substances and other important life processes in the body. Normal lipid metabolism plays an important role in maintaining the body's homeostasis, however, T2DM is often accompanied by disorders of lipid metabolism. Hyperglycemia and hyperlipidemia mostly coexist, which are mutually causal and jointly promote the development of diseases. In the IR state, the tissue's ability to utilize glucose is reduced, so it will mobilize fat lipolysis to release FFA and glycerol into the blood to supply energy to the body. Defective lipid metabolism promotes hyperlipidemia and ectopic lipid accumulation [60], and lipid overload in non-adipose tissue is associated with impairment of insulin signaling pathways and subsequent reduction in muscle glucose uptake, thereby resulting in muscle IR [61–63]. Muscle IR is directly associated with an increase in hepatic de novo-lipogenesis and subsequent increases in circulating triglycerides, very-low-density lipoprotein cholesterol, as well as a decrease in high-density lipoprotein cholesterol [64]. In this experiment, T2DM rats showed increased serum lipid profiles, involving DEPs ApoA1, ApoA4, ApoC1 and ApoC2, participating in cholesterol metabolism, fat digestion and absorption, and vitamin digestion and absorption signaling pathways, also the results of GO enrichment analysis were all correlated with lipid metabolism. This is consistent with the reported literature. Diabetic dyslipidemia among patients with T2DM is very common (prevalence of 72–85%) [65], it is characterized by moderate increases of LDL-C, elevations of TGs, low HDL-C, and small-dense (atherogenic) LDL-C particles and commonly related to insulin resistance [66]. Abnormal cholesterol metabolism, including low intestinal cholesterol absorption and elevated cholesterol biosynthesis, played an important role in metabolic syndrome, obesity, and diabetes [67]. Overexpression of SREBP2, which increases both cholesterol biosynthesis and LDL-receptor-mediated uptake of cholesterol, caused a diabetic phenotype by compromising the development of β-cells and inhibiting both basal and glucose induced insulin secretion [68]. Vitamin digestion and absorption signaling pathway are also closely related to the occurrence and development of T2DM. Vitamin D deficiency was very common in patients with IR or T2DM [69]. Some studies have shown that vitamin K or vitamin D supplementation in patients with T2DM improves blood glucose status and lowers blood lipids, thereby reducing the risk of T2DM and its complications [70, 71]. Population-based studies have shown that vitamin level was closely related to lipid metabolism [72]. Vitamin D may affect lipid profiles by reducing intestinal absorption of lipids, endogenic lipid synthesis, stimulating lipolysis, and improving lipid metabolism [73, 74]. Vitamin E exerts its antioxidant effects by preventing lipid peroxidation [75, 76]. ApoA4 is the apolipoprotein component of chylomicron particles and regulates the efficiency of intestinal cellular and hepatic transcellular lipid transport [77]. ApoA4 levels are positively correlated with dietary fat content, and ApoA4 under HFD conditions may promote weight gain and adipose tissue fat storage by limiting the entry of dietary fatty acids into the distal intestine, thereby reducing the secretion of intestinal hormones that suppress appetite and increase basal energy expenditure [78]. Studies have shown that ApoA4 levels are elevated in DM patients and may be a potential biomarker for DM and diabetic nephropathy [79, 80]. Moreover, there are reports that insulin may promote ApoA gene transcription [81]. ApoA1 and ApoA4 are located on human chromosome 11q23 and regulated by common transcription factors, with a significant correlation between them [82]. ApoC is the apolipoprotein of chylomicrons and VLDL. The protein ApoC1 regulates the activities of various enzymes related to HDL metabolism. It is responsible for the activation of lecithin-cholesterol acyltransferase (LCAT) and the inhibition of hepatic lipase (LIPC). In addition, ApoC1 has an important effect on ApoB-mediated uptake of VLDL in the liver. Overexpression of ApoC1 leads to a large increase in serum cholesterol and triglycerides as a result of VLDL accumulation [83]. In normolipidemic subjects, ApoC2 was found to be predominantly distributed in HDL, whereas in hypertriglyceridemic subjects it was predominantly distributed in VLDL and LDL [84]. As an essential activator of lipoprotein lipase (LPL), ApoC2 plays an important role in lipoprotein metabolism, while overexpression of ApoC2 inhibits LPL activity [85, 86]. In addition, ApoC2 inhibits ApoE- and ApoB-mediated binding of lipoproteins to LDL-R and LDL-R-associated proteins, thereby impeding the clearance of VLDL and chylomicron remnant [87, 88]. We observed that the expression level of ApoA1 and ApoA4 was down-regulated by MLE compared with the T2DM group, and the western blot assay further confirmed the results. Molecular docking also showed that the components of MLE have high binding activity to ApoA1 and ApoA4. This evidence suggests that ApoA1 and ApoA4 may be vital targets for MLE to treat T2DM by improving lipid transport and regulating lipid metabolism.

Due to the time and conditions, the biological replicates in this study were small and the amount of data generated was limited. In future studies, we will further expand the sample size, optimize the proteomics methods and equipment, and conduct more in-depth studies. Second, the pathologic results showed that skeletal muscle atrophy was attenuated in T2DM rats after MLE intervention, However, we could not conclude this because we did not formally assess the degenerative changes in muscle function. Future studies should investigate the inhibitory effect of MLE on the deterioration of skeletal muscle function in patients with T2DM. In addition, the validation experiments of this study were limited to the functional analysis of the histology and the level of protein expression regulation, we will further validate the functions of the targets by inhibitors or knockdown/overexpression methods in our future studies, and we will further explore the clinical significance of the important targets in combination with clinical samples.

## Conclusions

This study demonstrated that MLE can significantly improve hyperglycemia and hyperlipidemia in T2DM rats, and the mechanism may be related to the regulation of lipid transport and improvement of lipid metabolism. ApoA1 and ApoA4 may be the key targets for the therapeutic effect of MLE. The exact mechanism remains to be further investigated and characterized, but overall, the present findings provide new insights into the molecular mechanisms of MLE anti-T2DM and provide evidence to support the possibility of MLE as a therapeutic candidate for T2DM.

### Supplementary Information

**Additional file 1.**

**Additional file 2.**

## Data Availability

The datasets used and/or analysed during the current study are available from the corresponding author on reasonable request.

## Abbreviations

ADP: Adiponectin
ApoA1: Apolipoprotein A-1
AUC: Area under the curve
BP: Biological process
CC: Cellular component
DEPs: Differentially expressed proteins
DM: Diabetes mellitus
FBG: Fasting blood glucose
FFA: Free fatty acids
GO: Gene Ontology
HbA1c: Glycated hemoglobin A1c
HDL-C: High-density lipoprotein cholesterol
HE: Histopathological examination
HFD: High-fat diet
HOMA-IR: Homeostasis Model of Insulin Resistance
IR: Insulin resistance
ITT: Insulin tolerance test
KEGG: Kyoto Encyclopedia of Genes and Genomes
LDL-C: Low-density lipoprotein cholesterol
LEP: Leptin
MF: Molecular function
MLE: Mulberry leaf extract
OGTT: Oral glucose tolerance test
T2DM: Type 2 diabetes mellitus
TC: Total cholesterol
TCM: Traditional Chinese medicine
TG: Triglyceride
TMT: Tandem mass tag
PPI: Protein–protein interaction
VLDL: Very-low-density lipoprotein
