# Supplementary figures and images for: Identifying potential therapeutic targets of mulberry leaf extract for the treatment of type 2 diabetes: a TMT-based quantitative proteomic analysis
Source: BMC Complement Med Ther. 2023 Sep 4;23:308. doi: 10.1186/s12906-023-04140-3 (PMC10476348; doi:10.1186/s12906-023-04140-3)

## REPEAT 1

Tubulin

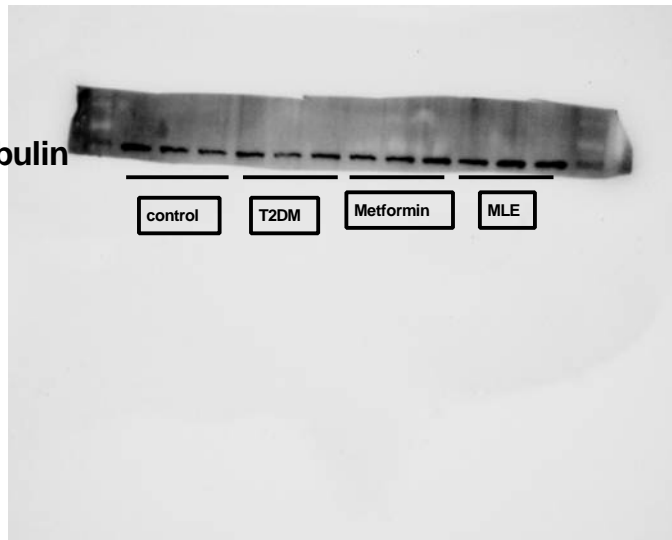

## REPEAT 2

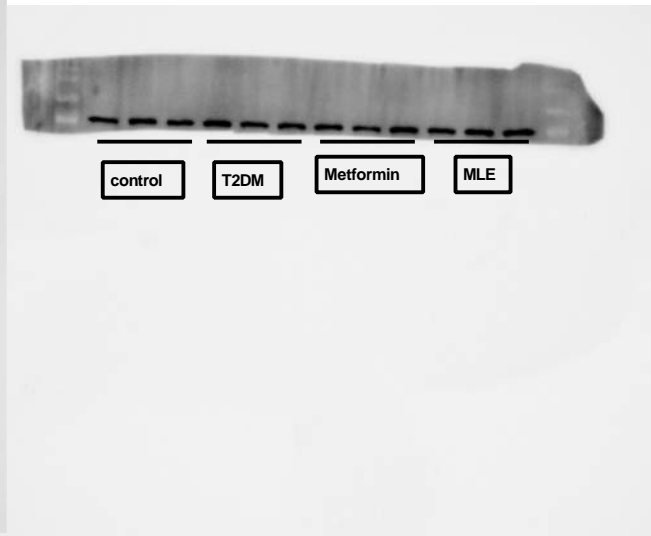

## REPEAT 3

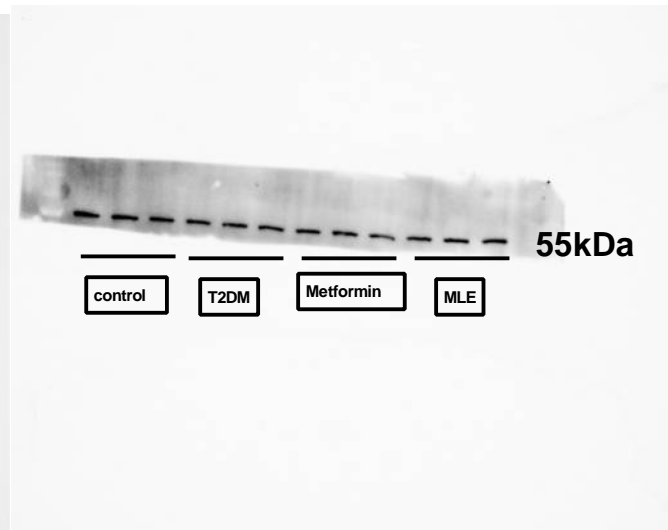

REPEAT 1

REPEAT 2

REPEAT 3

ApoA4

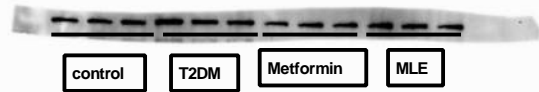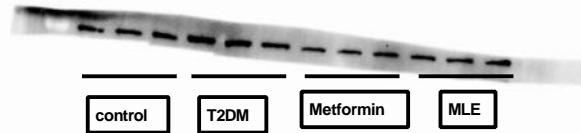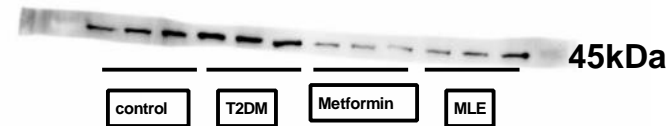

REPEAT 1

REPEAT 2

REPEAT 3

ApoA1

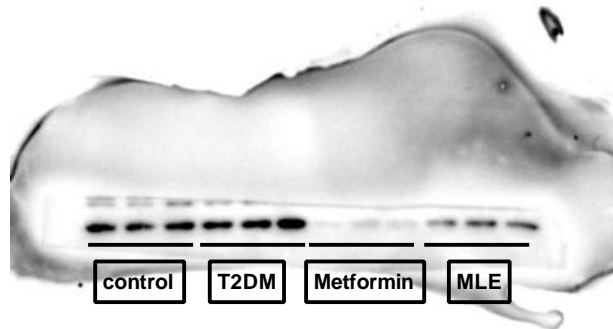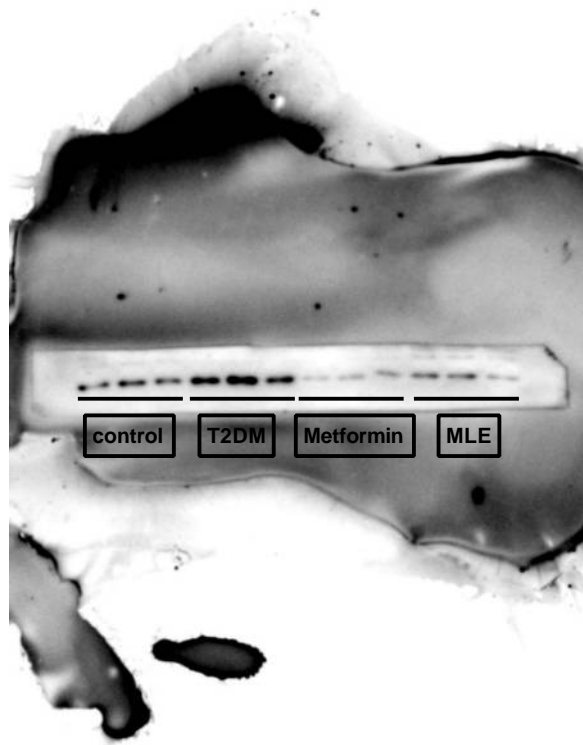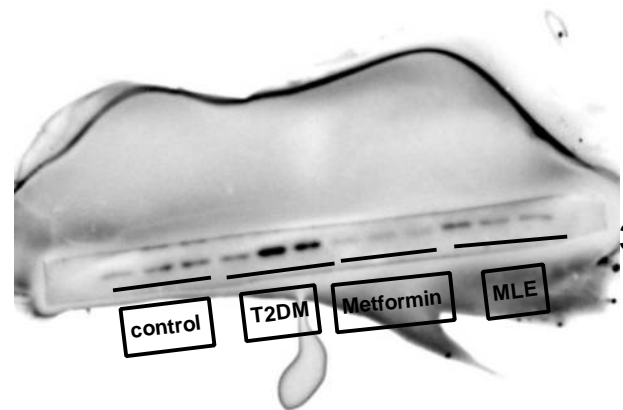

35kDa

Supplement: Supplementary file 1 — Additional file 1. [file 12906_2023_4140_MOESM1_ESM.pdf]
